# Balancing strategies in vaccination and mask-wearing for Chinese medical staff in post-COVID-19 era: a modelling study and cost-effectiveness analysis

**DOI:** 10.7189/jogh.15.04283

**Published:** 2025-09-26

**Authors:** Yiyu Hu, Meng Jia, Ying Jiang, Rui Zhao, Shu Su

**Affiliations:** 1 Department of Epidemiology and Biostatistics, The Second Affiliated Hospital of Chongqing Medical University, Chongqing, China; 2 Department of Travel Health, Chongqing International Travel Healthcare Centre (Port Outpatient of Chongqing Customs), Chongqing, China; 3 Shenzhen Hospital of Southern Medical University, Shenzhen, Guangdong, China

## Abstract

**Background:**

After the comprehensive easing of COVID-19 restrictions in China in December 2022, the emergence of additional virus variants and repeat infections has garnered increased attention, particularly among key populations such as medical staff. Our study aims to evaluate various combinations of vaccination and mask-wearing strategies to identify the optimal strategy for the post-COVID-19 era.

**Methods:**

A Markov model simulated a cohort of 100 000 Chinese medical staff five years after the complete lifting of epidemic restrictions. The health care system’s perspective was adopted, and parameters were sourced from Chinese government-released data and relevant literature. Strategies with varying vaccination coverage (0/25/50/75%) and mask-wearing coverage (0/30/60/90%) were considered. All costs were expressed as USD with a 3% annual discounting rate, and effectiveness was measured via quality-adjusted life year. Sensitivity analyses were performed to evaluate cost-effectiveness uncertainty.

**Results:**

At a willingness-to-pay threshold of one-time gross domestic product *per capita* in China (12 440 USD), the strategy of 90% mask-wearing and 75% vaccination coverage among medical staff was the most cost-effective (501 USD/quality-adjusted life year). This intervention reduced infection and mortality rates by 24.5% and 24.6%, while minimising health care costs with a cost per reversed infection of 1040 USD. Above 30% mask coverage, higher vaccine coverage further increased cost-effectiveness further. On the 16th day after lifting COVID-19 restrictions in the Markov model, there were 19 586 fewer symptomatic cases of first-time COVID-19 infection compared to the *status quo* (peak infections on that day = 22 293), with a reinfection circle around 197 days. Sensitivity analysis indicated that monthly mask costs is the most sensitive factor influencing the incremental cost-effectiveness ratio; beyond 6.2 USD/mo, the optimal strategy lost cost-effectiveness.

**Conclusions:**

In managing multiple waves of the epidemic, prioritising mask usage over vaccination is recommended for medical staff to achieve optimal cost-effectiveness. Exploring ways to extend vaccine efficacy duration would further enhance protection.

The emergence of the COVID-19 has prompted a global effort to defend against the virus [1]. From 2019 to 2022, the coronavirus has continuously mutated, resulting in diverse impacts on various regions. Given its varying degrees of severity and transmission, from the original virus to Omicron [2], China undertook decisive measures in December 2022, lifting critical restrictions, including the requirements of 14-day quarantine [3], physical distancing, home isolation, and mandatory mask-wearing, *etc*. [4,5]. Individuals were entrusted with safeguarding their own well-being, as the government redirected its focus toward developing new SARS-CoV-2 treatments [6,7]. Nevertheless, the challenge of COVID-19 persists, with the need for effective treatments still in progress [8] rendering individuals in China more susceptible to both initial infection and reinfection. Studies have shown that a notable proportion of recovered COVID-19 patients experience reinfection, highlighting the complexities of immunity and viral dynamics [9,10]. Moreover, reinfections often manifest with increased severity, particularly among individuals with underlying health conditions, posing significant risks of mortality [11,12]. The resurgence of SARS-CoV-2 variants has further compounded these challenges, necessitating continuous adaptation in public health strategies, especially among key populations [13].

Frontline medical staff are frequently exposed to patients during the post-COVID-19 era, amplifying their vulnerability to infection, reinfection, and increased mortality rates. Consequently, they experience substantial mental stress and a heavy workload in confronting dual challenges [14]. To mitigate the risk of infection and superinfection, certain nations and institutions have instituted comprehensive measures, including the provision of medical masks, face shields, other protective equipment, and tailored vaccination programmes for medical staff [15–17]. Governments typically provide resources and psychological support to help medical staff alleviate symptoms [18,19]. In the context of evolving vaccine strategies, including the development of nasal spray vaccines [20,21] and updated formulations to target emerging variants, questions remain regarding the effectiveness of these interventions in preventing reinfections among medical staff [22–24]. Yet, challenges persist, including the threat of reinfection due to asymptomatic carriers and diminished immunity among medical staff, which may delay the treatment of patients and contribute to further transmission [17,25]. Despite significant efforts to immunise vulnerable groups, there remains a notable gap in understanding reinfection risks and evaluating intervention efficacy among medical staff, particularly in the post-COVID-19 era.

This study employs an integrated deterministic and Markov model to assess the risk of infections and the potential for cyclic infections among medical staff in China. We hypothesise that high concurrent vaccination and mask-wearing coverage among medical staff will prove the most cost-effective strategy, reducing infections and mortality while remaining below the willingness-to-pay (WTP) threshold. By considering factors such as the working environment, the efficacy of combination strategies, and the viral transmission, this research aims to provide insights into the necessity and effectiveness of these combinations of vaccination and mask wearing strategies to manage COVID-19 reinfections among medical staff in China.

## METHODS

We developed an integrated transmission Markov model to evaluate the cost-effectiveness of different coverage rates of COVID-19 vaccines and traditional masks among medical staff in China, from a health care system perspective. The model was constructed to simulate the transmission of SARS-CoV-2 in a cohort of 100 000 Chinese medical staff using a decision-making Markov model (Figure S1 in the **Online Supplementary Document**). This model accounts for age-mixing patterns among medical staff in China after the relaxation of COVID-19 restrictions over five years. Disease progression, effectiveness of interventions (vaccines and mask coverage), and potential antiviral therapies were integrated into the model structure. Transitions between compartments were simulated through a stochastic chain binomial process.

### Parameters collection

This modelling study adopts the health care system’s perspective and relies on publicly available aggregated data (Table S1 in the **Online Supplementary Document**). The collected data rigorously reviewed and vetted to ensure their accuracy and relevance, enabling the model to simulate realistic scenarios for future outbreaks. Epidemiological indicators, infectivity, disease intervention and behavioural indicators, as well as the cost and utility values of testing and therapy, are the four main categories needed for this study. Epidemiological metrics encompass a broad range of critical COVID-19 indicators, including infection prevalence across different age groups, the prevalence of asymptomatic cases, the incidence rate of sequelae in treated patients, post-recovery prevalence, the persistence of antibodies in recovered individuals, and mortality rates among those who have received treatment for COVID-19. The rates of mask protection and timely vaccine serve as metrics for infectivity. Three behavioural indicators of disease intervention include the daily usage frequency of masks among medical staff, as well as the daily protective mask-wearing rate of medical staff. The costs associated with testing and treatment, vaccination, confirmatory novel coronavirus screening, cost of post-infection isolation and novel coronavirus therapy in various illness stages were collected. All costs were expressed in 2022 USD. As such, institutional review and informed consent are waived by the Institutional Review Board of the second affiliated hospital of Chongqing Medical University (Chongqing, China).

### Modelling scenarios

Since the full removal of COVID-19 restrictions in China, most of the population has stopped wearing masks in public and has not continued with vaccination. As the protective effects of previous vaccinations have likely waned, we assumed a 2% mask usage rate, consistent with pre-COVID-19 levels [26], and a 0% vaccination rate as the baseline (*status quo*) scenario for medical staff. We modelled a situation in which certain individuals would wear masks and receive vaccinations to target all age groups in the Chinese population during the COVID-19 prevalence. It is anticipated that all individuals will undergo a positive screening when the outbreak recurs. Based on the status quo, we simulated 15 combinations of vaccination and mask-wearing strategies by dividing all people in percentages: 75% vaccination +90% mask, 50% vaccination +90% mask, 25% vaccination +90% mask, 90% mask, 75% vaccination +60% mask, 50% vaccination +60% mask, 25% vaccination +60% mask, 60% mask, 75% vaccination +30% mask, 50% vaccination +30% mask, 25% vaccination +30% mask, 30% mask, 75% vaccination, 50% vaccination and 25% vaccination. Our base-case analysis focused on standard surgical masks, which reflect typical usage in general health care settings, with costs and efficacy parameters derived from their average performance metrics. We excluded N95 respirators from the primary model, as their use in China is typically restricted to high-risk aerosol-generating procedures. The model accounts for reinfection by transitioning individuals who recover from COVID-19 back to the susceptible status, allowing for repeated infections. Outcomes, including incident infections, reinfections, and deaths were quantified to evaluate costs and cost-effectiveness. Sensitivities and specificities of treatment methods were estimated using Chinese-specific data. The patients who fulfil the treatment standards will be vaccinated and hospitalised, and the specific treatment will be based on local guidelines and international standards [5,16].

### Cost-effectiveness analysis

To determine the cost-effectiveness of different screening strategies, we calculated the Incremental Cost Effectiveness Ratio (ICER), which represents the cost per quality-adjusted life-years (QALY) gained compared to the current practice (*status quo*). We adopted the WHO’s definition of cost-effectiveness, which states that the ICER should be less than three times gross domestic product (GDP) *per capita*. Specifically for China, we set the WTP threshold at one time GDP *per capita* (12 440 USD in 2022) following the WHO-CHOICE recommendation for cost-effectiveness benchmarks. This choice also aligns with recent empirical estimates suggesting lower thresholds may be more appropriate for China’s health care context [27]. We discounted future costs and QALYs at a rate of 3% per year. The ICER was determined through the ratio of the difference in health care costs to QALY by the corresponding combination strategies. The model was constructed using TreeAge Pro 2023 (TreeAge Software, LLC, Williamstown, Massachusetts, USA). and the analysis was reported according to the Consolidated Health Economic Evaluation Reporting Standards Statement 2022 (Table S2 in the **Online Supplementary Document**) [28].

### Sensitivity analysis

We implemented one-way deterministic sensitivity analyses for all parameters listed in Figure S1 and Table S1 in the **Online Supplementary Document**, testing each across its full evidence-based range (*e.g.* transmission probability ±20% from base values, vaccine efficacy 43.0–86.5%). Results were visualised through tornado diagrams identifying the 10 most influential parameters, ranked by their impact on ICER variability. Special attention was given to policy-relevant variables, including targeted evaluation of treatment coverage effects across 10–80% implementation rates. Parameter uncertainty ranges were derived from:

(1) systematic literature reviews for clinical parameters

(2) regional cost surveys for economic inputs (±30% variation)

(3) hospital-specific adherence data for behavioural factors.

This analytical approach ensured comprehensive assessment of both joint parameter uncertainties and individual parameter impacts on model outcomes.

## RESULTS

### Population impacts and cost-effectiveness of COVID-19 combination strategies and scenarios among medical staff

In the absence of prevention (*status quo*), the model was employed to simulate a cohort of 100 000 Chinese medical staff, who would experience 960 564 times of COVID-19 infection, resulting in 875 695 reinfections during five years after the complete lifting of epidemic restrictions in China. The investment cost and COVID-19-related medical expense for medical staff would amount to 275 928 677 USD per 100 000 people during five years, leading to 487 598 QALYs (**Table 1**).

**Table 1:** Effectiveness and cost-effectiveness of various COVID-19 intervention strategies in a cohort of 100 000 medical staff

Rank	Strategy	Cost (in USD)	Incr.cost (in USD)*	Eff†	Incr Eff‡	Incr C/E§		NMB¶	C/Eǁ	No. COVID cases	No. averted cases	No. Re-infected COVID cases	Ratio reinfection/first infection (in %)	No. fatal COVID cases	No. averted deaths	Cost per case averted (in USD)	Cost per fatal case averted		Cost per case averted (*vs*. *status quo*)	Cost per death averted (*vs*. *status quo*)
1	Status quo	275 928 677	-	487 598	-	-	5 789 784 505		566	960 564	-	875 695	91.2	1325	-	-	-		-	-
2	25% vaccination	275 740 516	−188 161	487 601	3	−54 002	5 790 016 011		566	960 304	259	875 436	91.2	1324	1	1 064 635	275 740 516		−725	−185 024
3	50% vaccination	275 479 879	−448 798	487 606	8	−54 045	5 790 336 606		565	959 944	620	875 075	91.2	1323	2	444 322	137 739 939		−724	−185 051
4	75% vaccination	275 045 593	−883 084	487 614	16	−54 140	5 790 870 500		564	959 341	1223	874 472	91.2	1320	5	224 894	55 009 118		−722	−185 097
5	30% mask	285 180 228	9 251 551	487 686	89	104 279	5 781 636 620		585	950 060	10 504	865 213	91.1	1312	13	27 149	21 936 941		881	702 416
6	25%vaccination +30% mask	284 802 261	8 873 584	487 693	96	92 807	5 782 100 350		584	949 292	11 271	864 446	91.1	1310	15	25 269	18 986 817		787	585 587
7	50%vaccination +30% mask	284 279 045	8 350 368	487 703	105	79 420	5 782 742 095		583	948 227	12 337	863 380	91.1	1307	18	23 043	15 793 280		677	466 588
8	75%vaccination +30% mask	283 407 703	7 479 026	487 718	121	61 827	5 783 810 305		581	946 445	14 119	861 599	91.0	1303	22	20 073	12 882 168		530	332 942
9	60% mask	292 580 335	16 651 658	487 847	250	66 657	5 776 240 480		600	931 806	28 758	847 002	90.9	1290	35	10 174	8 359 438		579	473 120
10	25%vaccination +60% mask	291 717 075	15 788 398	487 863	265	59 507	5 777 296 706		598	929 512	31 052	844 709	90.9	1286	40	9394		7 292 926	508	398 729
11	50%vaccination +60% mask	290 508 323	14 579 646	487 884	287	50 814	5 778 774 152		595	926 292	34 272	841 489	90.8	1279	46	8477		6 315 398	425	318 626
12	75%vaccination +60% mask	288 462 206	12 533 529	487 921	323	38 780	5 781 271 490		591	920 817	39 747	836 016	90.8	1269	56	7257		5 151 111	315	223 087
13	90% mask	260 196 251	−15 732 426	488 487	890	−17 678	5 816 587 709		533	774 392	186 172	689 674	89.1	1077	248	1398		1 049 178	−85	−63 499
14	25%vaccination +90% mask	256 939 716	−18 988 961	488 547	949	−20 005	5 820 581 522		526	764 027	196 537	679 313	88.9	1061	264	1307		973 257	−97	−71 885
15	50%vaccination +90% mask	252 395 712	−23 532 965	488 629	1031	−22 824	5 826 143 742		517	749 522	211 042	664 812	88.7	1038	287	1196		879 428	−112	−81 988
16	75%vaccination +90% mask	244 779 422	−31 149 255	488 764	1166	−26 707	5 835 442 754		501	725 098	235 466	640 395	88.3	1000	325	1040		753 167	−132	−95 740

C/E – cost-effectiveness, Incr. Eff. – incremental effectiveness, Incr. cost – incremental cost, Incr C/E – incremental cost-effectiveness, NMB – net monetary benefit

*Incr. cost in USD. The additional cost associated with a new intervention or strategy compared to an alternative or baseline.

†The measure of the health outcome or benefit of an intervention.

‡The additional effectiveness gained from the new intervention compared to the baseline or alternative.

§The ratio of the incremental cost to the incremental effectiveness.

¶The overall value of an intervention, calculated by subtracting the cost from the value of the effectiveness.

ǁThe ratio of the total cost to the total effectiveness.

For the different assumed scenarios, ICERs for various strategies in **Table 1** were calculated by comparing each strategy with the status quo. All strategy ICERs fall within the WTP. Among all strategies, the effectiveness of vaccination alone (75% vaccine 487 614 QALYs) was even lower than that of wearing masks alone (60% mask 487 847 QALYs). Among all sole vaccination strategies, the cost/effectiveness corresponding to the coverage of 25, 50, and 75% vaccinations were 566 USD/QALY, 565 USD/QALY and 564 USD/QALY, respectively, which was all significantly lower than that of 30% mask coverage only (585 USD/QALY). In addition, the cost/effectiveness for the strategy combining mask-wearing and vaccination (25% vaccination coverage and 30% mask coverage) was 584 USD/QALY, resulting in 768 less initial infections and reinfections compared to the 30% mask-only strategy. This indicates that the combined strategy of mask-wearing and vaccination was more effective at preventing the virus than relying solely on masks, with a certain percentage of individuals wearing masks. Moreover, when a certain proportion of the population wore masks (≥30% mask coverage), higher vaccination rates corresponded to increased cost-effectiveness. However, when there were sufficient individuals wearing masks, the strategy of high mask coverage alone proved to be more beneficial in terms of ICER compared to lower mask coverage with higher vaccination rates. Specifically, the cost-effectiveness for 90% mask coverage (533 USD/QALY) was higher than for the combined strategy of 75% vaccination coverage and 90% mask coverage (501 USD/QALY).

Among all the strategies, the most cost-effective was the strategy of 75% vaccination coverage and 90% mask-wearing coverage. If this combination strategy was applied among medical staff compared to the *status quo*, the number of infections could be reduced by 235 466 cases, the number of repeat infections by 235 299, and the number of deaths by 325 per 100 000 medical staff. Implementing a strategy with the coverage of 75% vaccination and the coverage of 90% mask-wearing achieved the highest cost-effectiveness, leading to a 24.6% reduction in mortality and a subsequent decrease in the number of deaths and overall health care expenses. The cost per infection reversed with this combination was 1040 USD which was the least expensive of all the combinations, which reversed 235 466 more infections and was the most cost-effective in relative terms. The second-lowest cost was attributed to the strategy of 50% vaccination combined with 90% mask-wearing coverage (**Table 1**).

### Estimation of COVID-19 trends among medical staff for five years

**Figure 1:**
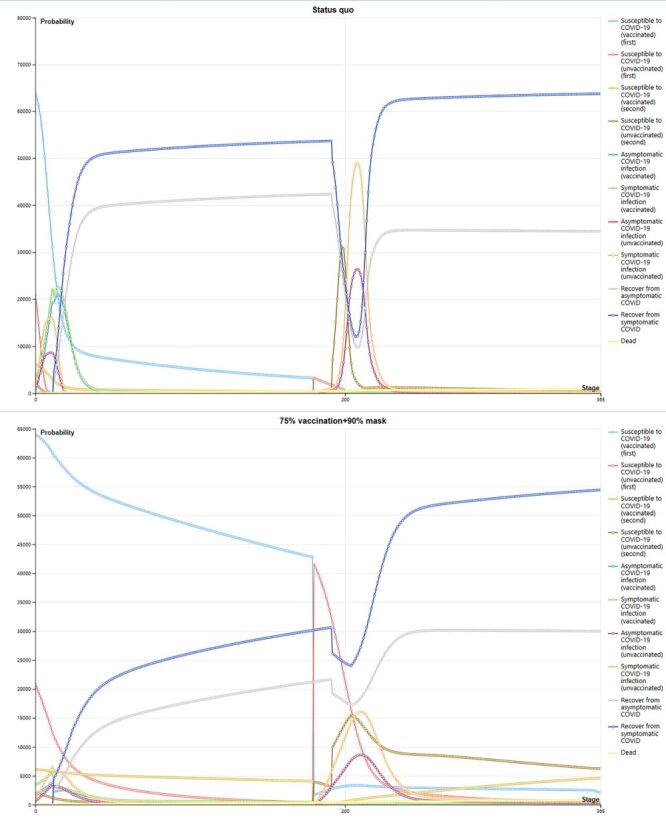
**Panel A.** Temporal population infections in *status quo*: trends in infections and recoveries among 100 000 medical staff over a one-year period under the *status quo*. **Panel B.** Temporal population infections with the combination of 75% vaccination and 90% mask coverage: trends in infections and recoveries among 100 000 medical staff over a one-year period under 75% vaccination and 90% mask-wearing coverage.

Given the consistent nature of the modelling scenario across years, we have carefully analysed the first year after lifting the restrictions. In a one-year Markov model simulating a cohort of 100 000 Chinese medical staff, the number of recovered symptomatic COVID-19 patients raised sharply around day 12, peaks near day 37, stabilised with a slight increase until approximately day 37–191, reached the nadir on day 207, then swiftly ascended to another peak and stabilised (around day 228). The overall trend for asymptomatic infections mirrors that of symptomatic cases, but asymptomatic patient recoveries declined compared to symptomatic cases, peaking near day 191 (42 339), rapidly declining, reaching a minimum around day 208. Towards the end of the year, symptomatic cases stabilised at around 63 000 cases, while recoveries for asymptomatic cases follow a linear trend, stabilised at approximately 34 000 cases (**Figure 1**, Panel A). Overall, the prevalence of infection was higher under *status quo* conditions, as indicated by the number of recoveries, resulting in a greater medical burden.

Under conditions of 75% vaccination and 90% mask usage coverage, symptomatic patient recoveries can peak at around 54 000 cases. The overall trend primarily comprised two phases: a relatively gradual increase followed by a decline around day 192, reaching a trough (day 204) and then a subsequent rise, characterised by rapid initial growth followed by gradual increase. Similarly, the trend for asymptomatic infections mirrors that of symptomatic cases. In such circumstances, the overall situation showed relative improvement, with less pronounced troughs in patient recovery rates and an increase in the susceptible population who have been vaccinated but remain uninfected. Initially, the peak susceptible population after the first vaccine dose reaches 64 000 cases. Overall, among the vaccinated population, both asymptomatic and symptomatic infection cases exhibit a declining trend (**Figure 1**, Panel B).

**Figure 2:**
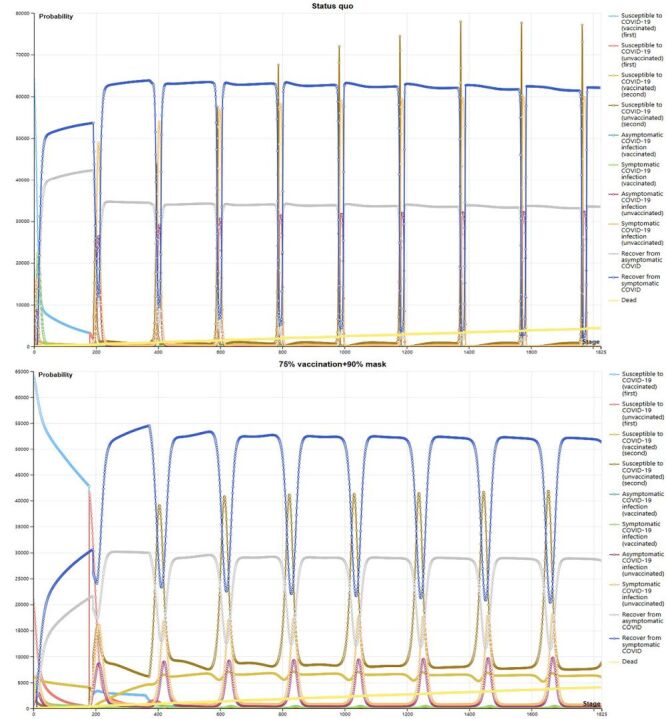
**Panel A.** Temporal population infections in *status quo*: trends in infections and recoveries among 100 000 medical staff over a five-year period under the *status quo*. **Panel B.** Temporal population infections with combination of 75% vaccination and 90% mask coverage: trends in infections and recoveries among 100 000 medical staff over a five-year period under 75% vaccination and 90% mask-wearing coverage.

Similar results were also estimated in the five-year Markov model. The reinfection cycle was approximately 197 days during the five years. From the first day, the yellow line at the bottom of the graph shows a cumulative increase in deaths over time until day 1825, though the numbers are relatively small compared to the overall population. However, the *status quo* scenario had a 24.5% higher cumulative death rate than the optimal strategy (**Figure 2**; Table S3 in the **Online Supplementary Document**).

### Sensitivity analysis

**Figure 3:**
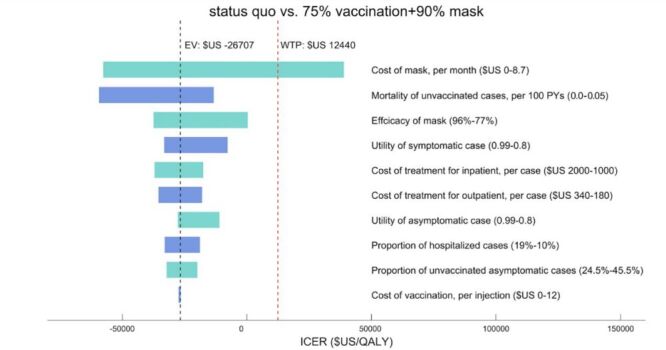
Tornado plot for specificity impact factors under conditions of 75% vaccination and 90% mask usage compared to the *status quo*.

We conducted one-way deterministic sensitivity analyses to assess the impact of parameter uncertainties and the robustness of our model, presented through tornado plots. Our findings indicated that preventive strategies, compared to the *status quo*, were generally robust to uncertainty across a broad spectrum of parameter values. Notably, the cost of masks per month, ranging from 0 to 8.7 USD, emerges as the most influential factor on the ICER. Notably, if the cost of masks exceeded 6.20 USD per month, the strategy would not be considered cost-effective under a one-time WTP threshold (**Figure 3**).

## DISCUSSION

Our study assesses the epidemiological impacts and cost-effectiveness of COVID-19 combination strategies among medical staff during the post-epidemic era. The findings indicate that increasing the coverage rates of mask-wearing, rather than vaccination alone, enhances cost-effectiveness and improves population protection. Given the diverse individual constitutions and varying immune responses to viral infections, the incidence of COVID-19 can differ. Accounting for these variations, we found that a combination of 75% vaccination and 90% mask usage is still the most cost-effective strategy within the range of disease incidence during the post-epidemic era.

Vaccination is universally recognised as an important measure in reducing transmission and infection rates. Previous research has demonstrated that vaccines are particularly cost-effective for high-risk populations [29], consistent with our findings. China's extensive and free vaccination campaign, coupled with periodic booster shots during the COVID-19 pandemic, has been noteworthy. One study reported a high willingness to pay for vaccination in China, with an average of 36.8 USD per person and a median of 14.5 USD, indicating strong vaccine acceptance [30]. Vaccination has also been found to lower the risk of long-COVID infection [31]. In a study by Strain WD et al., 72.4% of 812 patients with long-COVID experienced an increase in mild cases and a decrease in severe cases after vaccination [32]. Furthermore, vaccination administered approximately three months before a new COVID-19 infection has been shown to reduce long-COVID symptoms and effectively ameliorate symptoms in patients who have received more than one vaccine dose [31,33]. An Italian retrospective study underscores the significantly improved efficacy of mRNA vaccines, especially among high-risk individuals. The study suggests administering booster doses to high-risk individuals approximately six months after their initial vaccination [34,35]. Apart from the recurrent vaccination requirement due to the swiftly changing virus strains, we are continually innovating new vaccines for implementation, such as nasal spray vaccines [36]. In contrast, vaccine rejection was always due to diminished trust in medical institutions [37]. Therefore, it remains essential to encourage higher vaccination coverage rates.

Our research has demonstrated that the cost-effectiveness of mask-wearing alone significantly surpasses that of vaccination alone as a combination strategy. This finding is consistent with a study conducted in the USA, which showed that mask-wearing offers greater cost-effectiveness both before and after vaccination [38]. When vaccine efficacy diminishes, particularly in cases involving reduced immunity or escape variants, the cost-effectiveness of masks is further amplified [39]. As the rapid mutation of the virus can reduce the effectiveness of vaccination, masks always act as a physical barrier. COVID-19 shares transmission patterns like other respiratory pathogens, and mask usage reduces the occurrence of acute respiratory infections [40]. Additionally, appropriate mask usage effectively prevents droplet transmission, thereby curtailing the spread of the virus [41].

Nevertheless, mask-wearing can affect respiratory-hemodynamic parameters [42]. Extended mask-wearing, especially with FFP2 masks, results in slower, deeper respiration in younger and middle-aged individuals, while older individuals may experience breathing resistance or rapid respiration. Individuals with compromised cardiopulmonary function, such as those with chronic obstructive pulmonary disease, should avoid prolonged use of medical protective masks and prioritise vaccination. Healthcare workers may encounter adverse reactions from long-term mask use, including respiratory difficulties, discomfort at contact points, skin damage, and allergies [43]. Despite the potential adverse effects on specific individuals, mask-wearing is critical and necessary during peak waves of infection, and in other situations, the duration of mask-wearing can be extended as needed. In short, formulating a detailed mask-wearing policy to ensure the lowest necessary mask-wearing rate for medical staff is essential.

Our study indicates that, given the limited availability of personal protective equipment and vaccines in economically underdeveloped regions, minimising respiratory transmission through increased mask-wearing rather than implementing more resource-intensive intervention could be prioritised as it substantially reduces infection and mortality rates. We recommend:

(1) tiered mask allocation by departmental risk level

(2) price ceilings for sustainable procurement

(3) contingency plans for supply shortages.

In more developed areas, advocating for both mask-wearing and vaccination significantly enhances the likelihood of survival. Generally, a reduction in mask costs or an improvement in mask accessibility could encourage comprehensive mask-wearing among health care workers, leading to significant decreases in infection and mortality rates. However, it is essential to consider the implications of mask-wearing for high-risk groups. Prolonged mask-wearing may have adverse effects on these populations, such as discomfort or other health issues. Therefore, health care policies need to adapt by offering tailored guidance and support to ensure that these groups can effectively manage and mitigate potential risks. Furthermore, receiving two or more doses of the vaccine can further decrease the risk of viral infection and alleviate the symptoms of long-term COVID-19. Given the multitude of COVID-19 variants, although several vaccine types have been developed, continued research and development of new vaccines will likely be necessary as the situation evolves.

Several limitations should be noted in this study. First, our analysis only considered the average protective effectiveness of masks. According to previous studies [44,45], surgical masks and respirators offer the highest effectiveness, although their production is limited. Level 3 surgical masks exhibit superior filtration efficiency, capable of filtering over 98% of particles of 3.0 µm in size. In real health care settings, proper mask usage and comprehensive protective measures among health care workers may lead to even lower infection rates. However, due to China's vast geographical expanse, county hospitals and township clinics in underdeveloped regions may face resource constraints, resulting in limited access to surgical masks. Consequently, there is a possibility of substituting disposable masks or reusing them, which can potentially impact health care worker infection rates. Another limitation is the use of the health care system perspective rather than a broader societal perspective. While this approach is consistent with current pharmacoeconomic guidelines in China, it may overlook important indirect costs and benefits. Future research could incorporate these factors using a societal perspective, possibly by integrating productivity loss estimates, participants time costs, and broader public health externalities into the model. Third, while our model provides valuable insights into the cost-effectiveness of mask and vaccine coverage strategies for medical staff, it has not yet been formally validated against real-world outcomes due to the lack of available post-policy implementation data. Future studies should aim to validate model projections using empirical surveillance or administrative data when available. Fourth, although infection trends were modelled using a reinfection cycle to reflect the periodic nature of COVID-19 transmission, our model does not explicitly incorporate seasonal parameters such as climate-related factors or population mobility patterns. Incorporating such seasonal dynamics could further improve the model's precision and applicability. Finally, we did not account for individual differences in immunity and symptom changes. Repeated infections during subsequent waves of the pandemic and decrease in effectiveness of vaccination may result in more severe consequences for patients.

## CONCLUSIONS

Our findings suggest that even at the lowest coverage rates (25%) of masks and vaccinations for medical staff, these combination strategies are still cost-effective compared with no combination strategies. Wearing masks alone was more cost-effective than relying solely on vaccination to prevent COVID-19. In less-developed areas, where resources may be lacking, recommending mask usage is a practical strategy, although it is important to consider the potential adverse effects of prolonged mask-wearing. Promoting mask-wearing and vaccination concurrently in economically advanced regions can ensure comprehensive protection. Future research should focus on enhancing the effectiveness and duration of vaccines.

## Additional material


Online Supplementary Document
