# Supplementary material for: Balancing strategies in vaccination and mask-wearing for Chinese medical staff in post-COVID-19 era: a modelling study and cost-effectiveness analysis
Source: J Glob Health. 2025 Sep 26;15:04283. doi: 10.7189/jogh.15.04283 (PMC12491910; doi:10.7189/jogh.15.04283)
Supplement: Online Supplementary Document [file jogh-15-04283-s001.pdf]

Supplement to: Hu Y, Jia M, Jiang Y, Zhao R, Su S. Balancing strategies in vaccination and mask-wearing for Chinese medical staff in post-COVID-19 era: a modelling study and cost-effectiveness analysis. J Glob Health. 2025;15:04283.

Figure S1 Markov model of COVID-19 infection and reinfection among medical staff

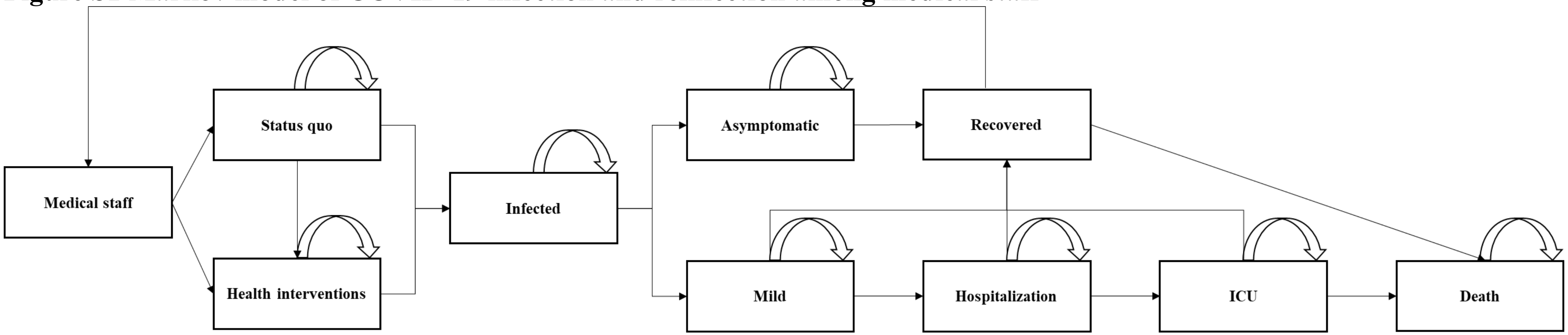

We developed a decision-analytic Markov model to simulate the disease progression of SARS-CoV-2 infection in a designated initial cohort of 100,000 medical staffs over a period of 5 years. The model consisted of 9 health states depicting varied disease progression of COVID-19 **(Figure S1)**. We defined the nine diseases states as follows. After being infected by SARS-CoV-2 strains, a fully vaccinated individual may progress through the following health states.

- Health interventions: the vaccine efficacy and mask efficacy
- Asymptomatic: cases who never developed symptoms ever throughout the course of their disease
- Mild/moderate: cases without pneumonia and cases with mild pneumonia
- Hospitalization: cases who developed dyspnoea and/or hypoxemia and managed in a hospital but not requiring intensive care unit (ICU)
- ICU: cases who developed respiratory failure, and/or septic shock, and/or multiple organ dysfunction/failure and managed in an ICU; some of them who recuperated from critical disease need to go through the recuperation stage-remaining in the hospital or other health care facility
- Recovered: cases who recovered from infection stages; we assumed that a recovered individual could not be reinfected for the next 180 days (similar to the short-term vaccine protection)
- Death: COVID-19 related death

Table S1 Model parameters for cost-effectiveness analysis of COVID-19 balancing strategy for medical staff in China

Parameter	Baseline(range)	Reference
Transmission coefficient, β	0.09 (0.01-0.10) The per-act transmission probability while in contact with infected individuals with symptoms (±20%)	[1, 2]
Prevalence of COVID-19 among the general population	5.13%-7.08%	[3]
Prevalence of COVID-19 among the medical staff	4.7%-27.3%	[4-6]
Cost (USD)		
Protection		
Nucleic acid test, per test	2.11 (1.48-2.74)	[7]
Masks, monthly	6.73 (1.26-8.75)	[7]
Vaccination, per person	9.50 (6.65-12.35)	[8]
Isolation gown, per unit	2.50 (2.0-3.0)	[8]
Gloves, per pair	0.2 (0.1-0.3)	[8]
Treatment for symptomatic infected patients		
Non-hospitalized patients, per person	264.0 (184.8-343.2)	Government report
Hospitalized patients, per person	1470.0 (1000.0-2000.0)	Government report
Number of people contacted by medical staff		
Patients, daily	6.50 (4.55-8.45)	[9]
Colleagues, daily	13.60 (9.52-17.68)	[9]
Hospitalization of symptomatic infected patients	0.15 (0.10-0.19)	[10]
Vaccine effective time, days	180 (126-234)	[11]
Vaccination rate in initial state	0.7540 (0.5278-0.9802)	[12]
Proportion of infected asymptomatic patients		
Vaccinated patients	0.4810 (0.3367-0.6253)	[10]
Unvaccinated patients	0.3500 (0.2450-0.4550)	[10]
Mortality		
Caused by COVID-19, annually	0.0630 (0.0441-0.0819)	[13]
Adult, annually	0.0063 (0.0044-0.0082)	[14]
Protection rate		
Masks	0.87 (0.77-0.96)	[15]
Vaccines	0.8650 (0.3890-0.9840)	[16]
Recovery rate after infection, annually	0.9370 (0.6559-0.9900)	[13]
Prevalence of COVID-19	0.0700 (0.0140-0.3120)	[17]
Average utility		
Asymptomatic infected patients	0.97 (0.80-0.99)	[18]
Mild/moderate symptomatic infected patients	0.94 (0.80-0.99)	[18]
Hospitalization infected patients	0.13 (0.09, 0.19)	[19, 20]
ICU patients	0.41 (0.27, 0.56)	[19, 20]
Death	0	[19, 20]

Table S2 Consolidated Health Economic Evaluation Reporting Standards (CHEERS) Checklist

Items to include when reporting economic evaluations of health interventions
The ISPOR CHEERS Task Force Report, Consolidated Health Economic Evaluation Reporting Standards (CHEERS)—Explanation and Elaboration: A Report of the ISPOR Health Economic Evaluations Publication Guidelines Good Reporting Practices Task Force, provides examples and further discussion of the 24-item CHEERS Checklist and the CHEERS Statement. It may be accessed via the Value in Health or via the ISPOR Health Economic Evaluation Publication Guidelines –CHEERS: Good Reporting Practices webpage:
http://www.ispor.org/TaskForces/EconomicPubGuidelines.asp

		Reporting Item	Page Number
Title			
	#1	Identify the study as an economic evaluation or use more specific terms such as "cost-effectiveness analysis", and describe the interventions compared.	1
Abstract			
	#2	Provide a structured summary of objectives, perspective, setting, methods (including study design and inputs), results (including base case and uncertainty analyses), and conclusions	2
Introduction			
Background and objectives	#3	Provide an explicit statement of the broader context for the study. Present the study question and its relevance for health policy or practice decisions	3
Methods			
Target population and subgroups	#4	Describe characteristics of the base case population and subgroups analysed, including why they were chosen.	4
Setting and location	#5	State relevant aspects of the system(s) in which the decision(s) need(s) to be made.	4
Study perspective	#6	Describe the perspective of the study and relate this to the costs being evaluated.	4
Comparators	#7	Describe the interventions or strategies being compared and state why they were chosen.	4-5
Time horizon	#8	State the time horizon(s) over which costs and consequences are being evaluated and say why appropriate.	4-5
Discount rate	#9	Report the choice of discount rate(s) used for costs and outcomes and say why appropriate	5
Choice of health outcomes	#10	Describe what outcomes were used as the measure(s) of benefit in the evaluation and their relevance for the type of analysis performed	5
Meaurement of effectiveness	#11a	Single study-based estimates: Describe fully the design features of the single effectiveness study and why the single study was a sufficient source of clinical effectiveness data	N/A
Measurement of effectiveness	#11b	Synthesis-based estimates: Describe fully the methods used for identification of included studies and synthesis of clinical effectiveness data	5
Measurement and valuation	#12	If applicable, describe the population and methods used to elicit preferences for outcomes.	N/A

of preference based outcomes			
Estimating resources and costs	#13a	Single study-based economic evaluation: Describe approaches used to estimate resource use associated with the alternative interventions. Describe primary or secondary research methods for valuing each resource item in terms of its unit cost. Describe any adjustments made to approximate to opportunity costs	N/A
Estimating resources and costs	#13b	Model-based economic evaluation: Describe approaches and data sources used to estimate resource use associated with model health states. Describe primary or secondary research methods for valuing each resource item in terms of its unit cost. Describe any adjustments made to approximate to opportunity costs.	5
Currency, price date, and conversion	#14	Report the dates of the estimated resource quantities and unit costs. Describe methods for adjusting estimated unit costs to the year of reported costs if necessary. Describe methods for converting costs into a common currency base and the exchange rate.	4
Choice of model	#15	Describe and give reasons for the specific type of decision analytical model used. Providing a figure to show model structure is strongly recommended.	4<br>Figure S1-2
Assumptions	#16	Describe all structural or other assumptions underpinning the decision-analytical model.	4-5
Analytical methods	#17	Describe all analytical methods supporting the evaluation. This could include methods for dealing with skewed, missing, or censored data; extrapolation methods; methods for pooling data; approaches to validate or make adjustments (such as half cycle corrections) to a model; and methods for handling population heterogeneity and uncertainty.	5
Results			
Study parameters	#18	Report the values, ranges, references, and, if used, probability distributions for all parameters. Report reasons or sources for distributions used to represent uncertainty where appropriate. Providing a table to show the input values is strongly recommended.	Table<br>Table S1
Incremental costs and outcomes	#19	For each intervention, report mean values for the main categories of estimated costs and outcomes of interest, as well as mean differences between the comparator groups. If applicable, report incremental cost-effectiveness ratios.	5-6
Characterising uncertainty	#20a	Single study-based economic evaluation: Describe the effects of sampling uncertainty for the estimated incremental cost and incremental effectiveness parameters, together with the impact of methodological assumptions (such as discount rate, study perspective).	7
Characterising uncertainty	#20b	Model-based economic evaluation: Describe the effects on the results of uncertainty for all input parameters, and uncertainty related to the structure of the model and assumptions.	5-6

Characterising heterogeneity	#21	If applicable, report differences in costs, outcomes, or cost effectiveness that can be explained by variations between subgroups of patients with different baseline characteristics or other observed variability in effects that are not reducible by more information.	7
Discussion			
Study findings, limitations, generalisability, and current knowledge	#22	Summarise key study findings and describe how they support the conclusions reached. Discuss limitations and the generalisability of the findings and how the findings fit with current knowledge.	7-9
Other			
Source of funding	#23	Describe how the study was funded and the role of the funder in the identification, design, conduct, and reporting of the analysis. Describe other non-monetary sources of support	10
Conflict of interest	#24	Describe any potential for conflict of interest of study contributors in accordance with journal policy. In the absence of a journal policy, we recommend authors comply with International Committee of Medical Journal Editors recommendations	9

For consistency, the CHEERS Statement checklist format is based on the format of the CONSORT statement checklist
The ISPOR CHEERS Task Force Report provides examples and further discussion of the 24-item CHEERS Checklist and the CHEERS Statement. It may be accessed via the Value in Health link or via the ISPOR Health Economic Evaluation Publication Guidelines – CHEERS: Good Reporting Practices
webpage: http://www.ispor.org/TaskForces/EconomicPubGuidelines.asp

The citation for the CHEERS Task Force Report is: Husereau D, Drummond M, Petrou S, et al. Consolidated health economic evaluation reporting standards (CHEERS)—Explanation and elaboration: A report of the ISPOR health economic evaluations publication guidelines good reporting practices task force. Value Health 2013; 16:231–50. [21]

Table S3 Comparative analysis of COVID-19 infection dynamics and outcomes among 100,000 medical staff over five years under status quo and optimal strategy scenarios

Scenarios	Days	State	State	State	State	State	State	State	State	State	Outcome	Outcome	Outcome	Outcome	Outcome
		Susceptible to COVID-19 (vaccinated, first-infection)	Susceptible to COVID-19 (unvaccinated, first-infection)	Susceptible to COVID-19 (vaccinated, reinfection)	Susceptible to COVID-19 (unvaccinated, reinfection)	Asymptomatic COVID-19 infection (vaccinated)	Symptomatic COVID-19 infection (vaccinated)	Asymptomatic COVID-19 infection (unvaccinated)	Symptomatic COVID-19 infection (unvaccinated)	Dead	Cumulative cost	Cumulative QALY	Cumulative number of COVID cases	Cumulative number of re-infected COVID cases	Cumulative number of fatal COVID cases
**Status quo**	0	64014	20885	6108	1993	1847	3431	603	1119	0	1123196	273	4096	357	1
**75%vaccination +90% mask**	0	64014	20885	6108	1993	1847	3431	603	1119	0	264725	273	889	77	1
**Status quo**	365	0	0	0	561	0	0	110	205	879	50968732	99484	188289	103420	252
**75%vaccination +90% mask**	365	2092	755	4589	6251	64	69	340	630	810	43972962	99637	130971	49100	183
**Status quo**	730	0	0	0	785	0	0	160	297	1772	107961364	197807	383941	299072	524
**75%vaccination +90% mask**	730	25	15	6724	8531	67	73	467	866	1660	99230992	198122	296620	211957	411
**Status quo**	1095	0	0	0	954	0	0	192	355	2657	164227923	295263	577101	492232	792
**75%vaccination +90% mask**	1095	1	0	6503	8067	65	70	437	812	2502	153928140	295786	460554	375853	636
**Status quo**	1460	0	0	0	491	0	0	74	138	3534	220104919	391858	768924	684055	1059
**75%vaccination +90% mask**	1460	0	0	6887	23173	363	391	9626	17860	3295	203905887	392683	608307	523605	819
**Status quo**	1825	0	0	0	39	0	0	4	8	4405	275928677	487598	960564	875695	1325
**75%vaccination +90% mask**	1825	0	0	5977	8883	60	64	435	807	4085	244779422	488764	725098	640395	1000

Reference for supplementary document:

1. Zhang XS, Vynnycky E, Charlett A, De Angelis D, Chen Z, Liu W. **Transmission dynamics and control measures of COVID-19 outbreak in China: a modelling study**. *Sci Rep* 2021; 11(1):2652.
2. van Boven M, van Dorp CH, Westerhof I, Jaddoe V, Heuvelman V, Duijts L, et al. **Estimation of introduction and transmission rates of SARS-CoV-2 in a prospective household study**. *PLOS Computational Biology* 2024; 20(1):e1011832.
3. Organization WH. **Coronavirus (COVID-19) infection survey (positivity estimates): 30 June to 6 July 2022**. In; 2022.
4. Yang Y-P, Pan S-J, Zhang M-X, Chen H-X, Tung T-H. **The impact of COVID-19 pandemic on healthcare workers under the "Ten New Guidelines" in Taizhou, China**. *Preventive Medicine Reports* 2024; 37:102550.
5. Milambo JPM, Ndirangu J, Mangala S, Simba H, Kabego L. **Incidence of Coronavirus Disease 2019 (COVID-19) among healthcare workers during the first and second wave in the Democratic Republic of the Congo: a descriptive study**. *BMC Infectious Diseases* 2023; 23(1):519.
6. Baldi ME, Laghrissi A, Marso Z, Chellat FZ, Berraho M, Tachfouti N, et al. **Prevalence and associated factors of COVID-19 among Moroccan physicians: A cross-sectional study**. *PLOS ONE* 2022; 17(11):e0277157.
7. An X, Xiao L, Yang X, Tang X, Lai F, Liang XH. **Economic burden of public health care and hospitalisation associated with COVID-19 in China**. *Public Health* 2022; 203:65-74.
8. **The expenditure of COVID-19 vaccine in China is more than 120 billion yuan**. In. Chinese: The Central People's Government of the People's Republic of China; 2022.
9. **Statistical Bulletin on the Development of Health Care in China in 2021**. *Chinese Journal of Viral Diseases* 2022; 12(05):321-330.
10. Lin YF, Wu X, Li Y, Bian J, Li K, Jiang Y, et al. **Impact of combination preventative interventions on hospitalization and death under the pandemic of SARS-CoV-2 Omicron variant in China**. *J Med Virol* 2023; 95(1):e28335.
11. Liang XM, Xu QY, Jia ZJ, Wu MJ, Liu YY, Lin LR, et al. **A Third Dose of an Inactivated Vaccine Dramatically Increased the Levels and Decay Times of Anti-SARS-CoV-2 Antibodies, but Disappointingly Declined Again: A Prospective, Longitudinal, Cohort Study at 18 Serial Time Points Over 368 Days**. *Front Immunol* 2022; 13:876037.
12. Huang W, Shao X, Wagner AL, Chen Y, Guan B, Boulton ML, et al. **COVID-19 vaccine coverage, concerns, and preferences among Chinese ICU clinicians: a nationwide online survey**. *Expert Rev Vaccines* 2021; 20(10):1361-1367.
13. Xiong Q, Xu M, Li J, Liu Y, Zhang J, Xu Y, et al. **Clinical sequelae of COVID-19 survivors in Wuhan, China: a single-centre longitudinal study**. *Clin Microbiol Infect* 2021; 27(1):89-95.
14. **China Statistical Yearbook-2020**. Chinese: China Statistics Press; 2020.
15. Bundgaard H, Bundgaard JS, Raaschou-Pedersen DET, von Buchwald C, Todsen T, Norsk JB, et al. **Effectiveness of Adding a Mask Recommendation to Other Public Health Measures to Prevent SARS-CoV-2 Infection in Danish Mask Wearers : A Randomized Controlled Trial**. *Ann Intern Med* 2021; 174(3):335-343.

16. Dai L, Gao L, Tao L, Hadinegoro SR, Erkin M, Ying Z, et al. **Efficacy and Safety of the RBD-Dimer-Based Covid-19 Vaccine ZF2001 in Adults**. *N Engl J Med* 2022; 386(22):2097-2111.
17. WHO. **Number of COVID-19 cases reported to WHO**. In; 2024.
18. Zhang L, Lei J, Zhang J, Yin L, Chen Y, Xi Y, et al. **Undiagnosed Long COVID-19 in China Among Non-vaccinated Individuals: Identifying Persistent Symptoms and Impacts on Patients' Health-Related Quality of Life**. *J Epidemiol Glob Health* 2022; 12(4):560-571.
19. Cleary SM, Wilkinson T, Tamandjou Tchuem CR, Docrat S, Solanki GC. **Cost-effectiveness of intensive care for hospitalized COVID-19 patients: experience from South Africa**. *BMC Health Serv Res* 2021; 21(1):82.
20. **Global, regional, and national incidence, prevalence, and years lived with disability for 354 diseases and injuries for 195 countries and territories, 1990-2017: a systematic analysis for the Global Burden of Disease Study 2017**. *Lancet* 2018; 392(10159):1789-1858.
21. Husereau D, Drummond M, Petrou S, Carswell C, Moher D, Greenberg D, et al. **Consolidated Health Economic Evaluation Reporting Standards (CHEERS)--explanation and elaboration: a report of the ISPOR Health Economic Evaluation Publication Guidelines Good Reporting Practices Task Force**. *Value Health* 2013; 16(2):231-250.
